# Advances in Trop-2 targeted antibody-drug conjugates for breast cancer: mechanisms, clinical applications, and future directions

**DOI:** 10.3389/fimmu.2024.1495675

**Published:** 2024-11-01

**Authors:** Yujun Tong, Xiaobing Fan, Huan Liu, Tiantian Liang

**Affiliations:** ^1^ Department of Breast Center, Mianyang Central Hospital, School of Medicine, University of Electronic Science and Technology of China, Mianyang, China; ^2^ Department of Respiratory and Critical Care Medicine, Mianyang Central Hospital, School of Medicine, University of Electronic Science and Technology of China, Mianyang, China; ^3^ Mianyang Key Laboratory of Anesthesia and Neuroregulation, Department of Anesthesiology, Mianyang Central Hospital, Mianyang, China; ^4^ Department of Pediatrics, Mianyang Central Hospital, School of Medicine, University of Electronic Science and Technology of China, Mianyang, China; ^5^ Department of Pharmacy, Mianyang Central Hospital, School of Medicine, University of Electronic Science and Technology of China, Mianyang, China

**Keywords:** Trop-2, antibody-drug conjugates, breast cancer, Sacituzumab Govitecan, Datopotamab Deruxtecan, targeted therapy, combination therapy, tumor microenvironment 1

## Abstract

Breast cancer remains a leading cause of cancer-related deaths among women worldwide, highlighting the need for novel therapeutic strategies. Trophoblast cell surface antigen 2 (Trop-2), a type I transmembrane glycoprotein highly expressed in various solid tumors including all subtypes of breast cancer, has emerged as a promising target for cancer therapy. This review focuses on recent advancements in Trop-2-targeted antibody-drug conjugates (ADCs) for breast cancer treatment. We comprehensively analyzed the structure and mechanism of action of ADCs, as well as the role of Trop-2 in breast cancer progression and prognosis. Several Trop-2-targeted ADCs, such as Sacituzumab Govitecan (SG) and Datopotamab Deruxtecan (Dato-DXd), have demonstrated significant antitumor activity in clinical trials for both triple-negative breast cancer (TNBC) and hormone receptor-positive/HER2-negative (HR+/HER2-) breast cancer. We systematically reviewed the ongoing clinical studies of these ADCs, highlighting their efficacy and safety profiles. Furthermore, we explored the potential of combining Trop-2-targeted ADCs with other therapeutic modalities, including immunotherapy, targeted therapies, and small molecule inhibitors. Notably, Trop-2-targeted ADCs have shown promise in reprogramming the tumor microenvironment through multiple signaling pathways, potentially enhancing antitumor immunity. This review aims to provide new insights and research directions for the development of innovative breast cancer therapies, offering potential solutions to improve treatment outcomes and quality of life for breast cancer patients.

## 1 Introduction

Breast cancer is one of the most common cancers among women worldwide and is a leading cause of cancer-related deaths in women. According to the latest global cancer data from 2022, there were approximately 2.3 million new cases of breast cancer, accounting for 11.6% of all cancer cases. Among female cancer cases worldwide, breast cancer represents nearly one-fourth, and nearly one-sixth of all cancer-related deaths (1). Despite significant progress in the early diagnosis and treatment of breast cancer, advanced and metastatic breast cancer remain challenging to cure. Breast cancer is a heterogeneous disease with various molecular subtypes, including hormone receptor-positive (HR+), human epidermal growth factor receptor 2-positive (HER2+), and triple-negative breast cancer (TNBC) (2).Traditional treatment methods include surgery, radiotherapy, chemotherapy, and endocrine therapy (3). However, due to tumor heterogeneity and the presence of drug resistance, many patients have a limited response to existing treatment options. Therefore, there is an urgent need to develop new targets and drugs to improve the effectiveness of breast cancer treatment.

Trophoblast cell surface antigen 2 (Trop-2) is a cell surface glycoprotein that is expressed at relatively low expression in normal tissues but is highly expressed in various solid tumors, including all subtypes of breast cancer, where it plays a crucial role in tumor progression (4, 5). Studies have shown that downregulation of Trop-2 delays the growth of TNBC cells and tumors, while upregulation of Trop-2 is associated with various aggressive tumor characteristics, such as enhanced tumor growth, invasion, metastasis, and treatment resistance. This highlights its oncogenic significance in breast cancer (2, 6, 7). Therefore, it is a highly promising and ideal tumor target. Antibody-drug conjugates (ADCs) are a new class of targeted therapeutics that specifically bind to target antigens on the surface of tumor cells via antibodies, delivering potent cytotoxic drugs directly into tumor cells, thereby reducing damage to normal tissues (8). Researchers have combined the ideal target Trop-2 with the design concept of ADC drugs, creating Trop-2-targeted ADCs that have demonstrated clinical efficacy in various cancers (9, 10). Compared to traditional chemotherapy drugs, Trop-2-targeted ADCs enhance drug efficacy and reduce side effects by selectively attacking tumor cells with high Trop-2 expression. This strategy not only prolongs progression-free survival but also significantly improves overall survival rates. Currently, Sacituzumab Govitecan is one of the most representative Trop-2-targeting ADCs. Preclinical and clinical studies have demonstrated that Sacituzumab Govitecan exhibits significant efficacy and acceptable safety in treating refractory and metastatic breast cancer patients (11). In this review, we summarized the current research status of Trop-2-targeted ADC drugs in breast cancer, aiming to provide new references and research ideas for the development of new drugs for breast cancer.

## 2 Overview of ADC targeting Trop-2

### 2.1 Structure and mechanism of action of ADCs

The design concept of ADCs has a long history. Over 100 years ago, German scientist Paul Ehrlich was the first to propose the concept of “selective delivery of cytotoxic drugs to tumor cells.” Using the phrase “magic bullet”, he inadvertently unveiled antibody-drug conjugates, hoping that the drug could be delivered to a specific target by means of a guided system (12, 13). ADCs are primarily composed of three structural components: a monoclonal antibody, a cytotoxic agent (also known as the “payload” or “warhead”), and a linker (14). A complete ADC is an immunoconjugate formed by linking a monoclonal antibody to a cytotoxic agent via a linker.

To maximize the efficacy of tumor-targeted therapy and reduce toxicity, the selection of the three components of an ADC is critically important. Among these components, the monoclonal antibody and the cytotoxic agent are the main contributors to the antitumor effect. An ideal antibody should have minimal immunogenicity, high specificity, and strong binding affinity for the target antigen to ensure effective internalization and a long circulating half-life. The antigen on the tumor cell surface that specifically binds to the antibody is also crucial. An ideal target antigen should be uniformly and stably expressed on the surface of target cells, with low or no expression in healthy tissues, and should not shed or have minimal free antigen to limit off-target toxicity. The selection of the cytotoxic agent is critical; it should have a potent antitumor effect and be capable of exerting a cell-killing effect at picomolar levels when released from the ADC. Currently, the cytotoxic agents used in ADCs mainly include DNA-damaging agents, microtubule inhibitors, RNA polymerase inhibitors, and topoisomerase inhibitors. The selection of the linker is one of the key points in constructing ADC drugs, as it can directly affect the circulation stability, pharmacokinetics, and pharmacodynamics of the ADC molecules. ADC linkers are categorized into cleavable and non-cleavable types. Cleavable linkers rapidly break down in low pH or protease-rich lysosomal environments, releasing the payload, which, if membrane-permeable, can potentially exert a bystander effect to eliminate tumors. Non-cleavable linkers are inert in the body, offering higher stability. After entering tumor cells, they release cytotoxic molecules upon lysosomal degradation. However, amino acid residues remain attached to the linker and cytotoxic agent during degradation, making it difficult for these charged metabolites to diffuse across biological membranes, thus almost eliminating the bystander killing effect (15, 16).

**Figure 1:**
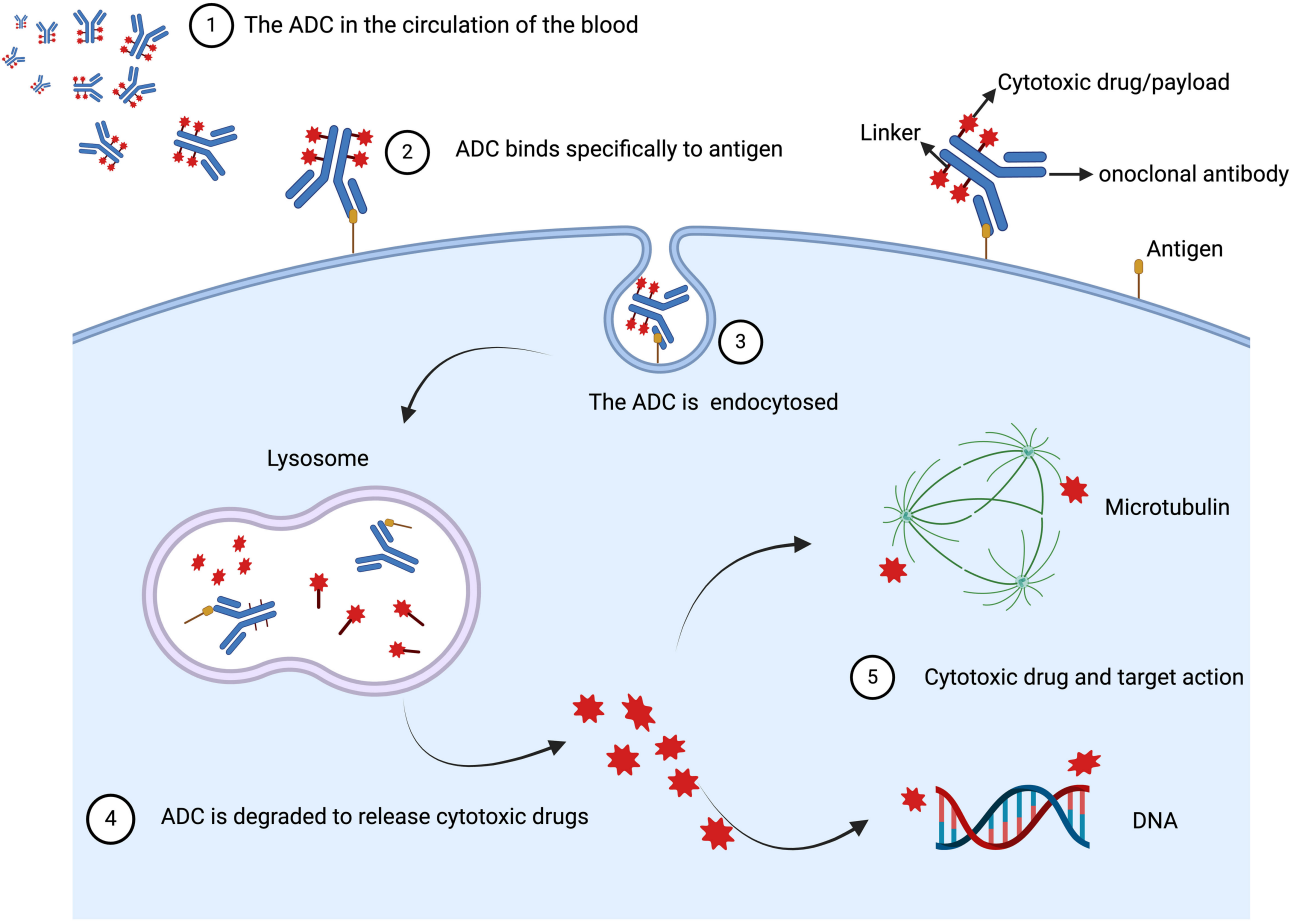
The basic mechanisms of action of ADC *in vivo*. ADC is composed of three distinctive parts: the antibody, linker, and cytotoxic drug/payload. After ADCs enter the bloodstream and reach the tumor site, the monoclonal antibody binds to the target antigen on the cell surface through its antigen-binding fragment, forming a complex that is then internalized by the tumor cell. In the tumor microenvironment, the linker within the lysosome is cleaved through proteolysis/acidity, leading to the release of the cytotoxic drug inside the tumor cell, which then disrupts microtubules or DNA, thereby exerting a potent cytotoxic effect on the tumor cells. Created with Liu, H. (2024) BioRender.com/r48l887.

After ADCs enter the bloodstream and reach the tumor site, the monoclonal antibody binds to the target antigen on the cell surface through its antigen-binding fragment, forming a complex that is then internalized by the tumor cell. In the tumor microenvironment, the linker within the lysosome is cleaved through proteolysis/acidity, leading to the release of the cytotoxic drug inside the tumor cell, which then disrupts microtubules or DNA, thereby exerting a potent cytotoxic effect on the tumor cells (9). Additionally, less polar payloads may permeate the cell membrane and, once released inside the tumor cell, can diffuse through the cytoplasm to the surrounding tumor environment and neighboring cells, exerting a bystander effect. This effect allows some ADCs to have broad activity even in cancers with low or heterogeneous expression of the target antigen (**Figure 1**) (17). Therefore, ADCs are a new class of targeted therapies for tumors. By leveraging the targeting properties of monoclonal antibodies, ADCs can significantly enhance the delivery of the attached payload while reducing the adverse effects of the drug. The development of ADCs has been very rapid, with third-generation ADCs now in development. First-generation ADCs had weak targeting ability, high immunogenicity, and instability of the linker, which could lead to the premature release of the cytotoxic agent and associated toxic reactions. Second-generation ADCs used non-cleavable linkers to avoid the issue of premature release of the cytotoxic agent before entering the target cell. Third-generation ADCs have addressed the major limitations of the first and second-generation ADCs through multiple technological advancements. First, the new generation ADCs use humanized or fully human antibodies, reducing drug resistance and immunogenicity, significantly improving patient tolerance and the drug’s target specificity (18). Second, the design of the linkers has been optimized. These new linkers stabilize the drug during transport in the bloodstream and minimize the risk of premature release. They are precisely activated in the tumor microenvironment, such as under low pH or in the presence of specific enzymes, thereby reducing systemic toxicity (19). Additionally, third-generation ADCs employ more potent and highly specific cytotoxic agents, such as modified auristatins or topoisomerase inhibitors, which can more effectively kill rapidly proliferating tumor cells. By optimizing the drug-to-antibody ratio (DAR), the release of the drug within the tumor is more balanced, ensuring higher therapeutic efficacy while reducing toxicity to normal tissues (20). In summary, these improvements allow third-generation ADCs to exhibit better clinical efficacy, lower side effects, and stronger therapeutic effects against drug-resistant tumors, offering superior clinical outcomes and safety (14).

### 2.2 Structure and pathway of Trop-2

**Figure 2:**
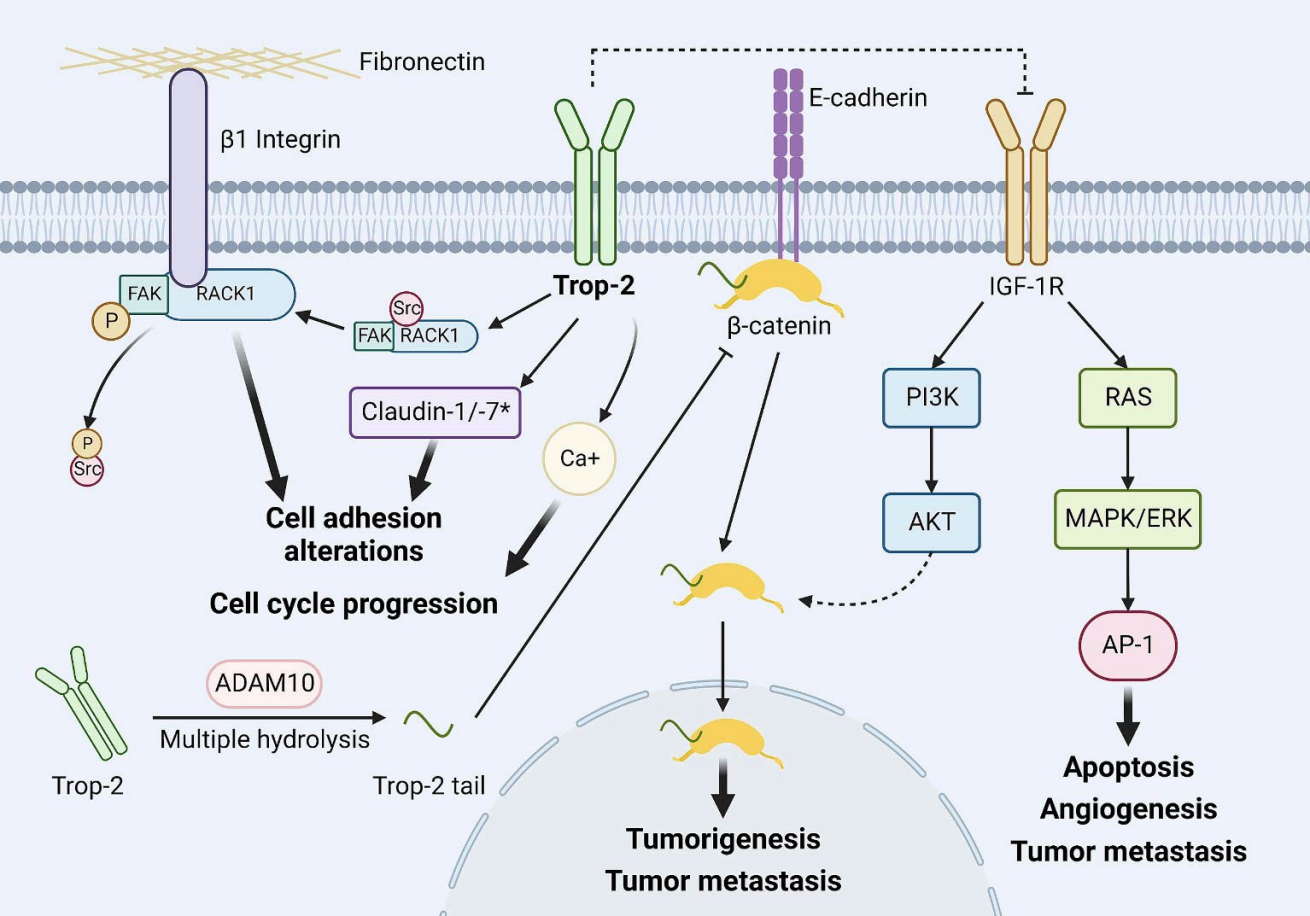
Major downstream events of Trop-2. Trop-2 inhibits the IGF-1R signaling pathway by binding to IGF-1 and inhibits apoptosis, angiogenesis, and tumor metastasis by downregulating transcription factor AP-1 levels via RAS-MAPK/ERK. The cleavage activated fragment of Trop-2 binds to β-catenin, causing it to dissociate from E-cadherin and translocate to the nucleus, initiating transcription of genes involved in tumor formation and tumor metastasis. Trop-2 activates the β1 integrin-RACK1-FAK-Src signaling axis, which regulates the adhesion of tumor cells to fibronectin. Trop-2- mediated Ca2+ release plays an important role in cell cycle progression. Copyright ^©^ 2023 Elsevier BV. Creative Commons (9).

Trop-2 was first identified in human trophoblast cells in 1981, and its antibody is considered useful for the diagnosis and treatment of choriocarcinoma and reproductive system diseases (21). Trop-2 is a cell surface glycoprotein encoded by the TACSTD2 gene and is a member of the TACSTD protein family. It is a single-pass transmembrane protein (a type I transmembrane cell surface glycoprotein), also known as epithelial glycoprotein-1 (EGP-1), tumor-associated calcium signal transducer-2 (TACSTD2), and gastrointestinal tumor-associated antigen 733-1 (GA733-1). The primary structure of Trop-2 consists of a 36 kDa peptide made up of 323 amino acids, comprising a cytoplasmic tail, an extracellular domain, a hydrophobic signal peptide, and a transmembrane region (22). With further research, the biochemical profile of Trop-2 has gradually become clearer. It is primarily expressed in epithelial cells. Under physiological conditions, Trop-2 plays a crucial role in embryonic development, placental tissue formation, and stem cell proliferation, with normally low expression levels. When Trop-2 is overexpressed, it acts as an oncogene, interacting with many key cellular signaling pathways in tumor cells, potentially promoting the proliferation, growth, invasion, and metastasis of various malignant tumors such as breast cancer, colon cancer, papillary thyroid carcinoma, and esophageal squamous cell carcinoma, among others, playing a critical role in cancer development (5, 7). Several key pathways have been identified (**Figure 2**) (9): (1) Trop-2 impedes the IGF-1R signaling pathway by forming a complex with insulin-like growth factor 1 (IGF-1). Additionally, it suppresses apoptosis, angiogenesis, and tumor metastasis by downregulating the transcription factor activator protein 1 (AP-1) via the RAS-MAPK/ERK pathway (23–27). (2) The cleaved active fragment of Trop-2 binds to β-catenin, causing it to dissociate from E-cadherin and translocate to the nucleus, where it initiates the transcription of genes involved in tumor formation and metastasis (24). (3) Trop-2 activates the β1 integrin-RACK1-FAK-Src signaling axis, which regulates the adhesion of tumor cells to fibronectin (28). (4) As a transmembrane calcium signaling protein, Trop-2-mediated calcium release plays a crucial role in cell cycle progression (23). Trop-2, as an oncogene, often plays multiple roles in tumor development. For example, researchers discovered in colorectal cancer that Trop-2, Na+/K+ ATPase, CD9, PKCα, and cofilin assemble into a membrane signaling super complex, driving the growth and invasion of colorectal cancer. In this study, it was found that Trop-2 binds to the cell membrane Na+/K+-ATPase, leading to an intracellular increase in Ca^2+^, which subsequently triggers the membrane translocation of PKCα, causing phosphorylation of the Trop-2 cytoplasmic tail. Trop-2, anchored to the membrane by PKCα and CD9, promotes this feed-forward signaling pathway. Trop-2 requires CD9 to recruit PKCα and cofilin-1 to the cell membrane. This induces malignant progression through proteolytic cleavage of E-cadherin, remodeling of the β-actin cytoskeleton, and activation of Akt and ERK (29). Therefore, Trop-2 is important in tumor cell self-renewal, proliferation, invasion, and transformation, and is closely associated with poor prognosis and recurrence of tumors, making it a molecular marker for assessing tumor malignancy and a potential therapeutic target.

Researchers have perfectly combined the design concept of ADC drugs with the specificity of the Trop-2 receptor, leading to the development of several new antitumor drugs. In April 2020, the first Trop-2-targeted ADC, IMMU-132, was approved for market release, establishing Trop-2 as a prominent target in tumor-targeted therapy and igniting a surge in the development of ADCs targeting this receptor.

## 3 Trop-2 and breast cancer

### 3.1 Trop-2 expression in breast cancer

The expression pattern of Trop-2 exhibits heterogeneity in almost all breast tumor tissue types. Researchers utilized microarray data from three relevant databases—ISP 1 gene expression dataset (n=149), METABRIC expression dataset (n=1992), and TCGA expression dataset (n=817)—to assess the expression of the TACSTD2 (Trop-2) gene. They screened for correlation between TACSTD2 gene expression and the expression of selected genes involved in the development of breast cancer. The results indicated that the TACSTD2 gene is expressed across all breast cancer subtypes, with a particularly wide expression range in luminal A and TNBC subtypes. It is also associated with the expression of genes involved in epithelial-to-mesenchymal transition, adhesion, and proliferation, thereby promoting breast cancer growth (30). Richard B. Mertens conducted immunohistochemical staining for Trop-2 on 94 untreated primary invasive breast cancers, including 25 luminal A-like, 25 luminal B-like, 19 HER2-like, and 25 triple-negative tumors. He found that Trop-2 was expressed at moderate to high levels in most cases across different molecular subtypes, with a wide range of expression (31). Immunohistochemistry (IHC) was used to detect Trop-2 protein expression in early-stage luminal-like breast cancer and to analyze its correlation with clinicopathological characteristics. It was found that Trop-2 is highly expressed in early-stage luminal-like breast cancer (94%) and that Trop-2 expression is not correlated with clinicopathological features such as patient age, histological subtype, pathological grade, Ki67, tumor size, lymph node status, or lymph node metastasis. Trop-2 expression is unaffected by clinicopathological characteristics (32).

Compared to other types of breast cancer, Trop-2 expression is highest in TNBC (31, 33). Researchers assessed Trop-2 expression through IHC on tumor samples from patients diagnosed with TNBC between 2000 and 2017. By analyzing the correlation between Trop-2 expression and baseline tumor characteristics, they found that higher Trop-2 expression was associated with apocrine histology, higher androgen receptor (AR) expression, ductal carcinoma *in situ* (DCIS), lymphovascular invasion (LVI), and lymph node involvement. However, Trop-2 expression was not associated with stromal tumor-infiltrating lymphocytes (sTIL), event-free survival outcomes, or the rate of pathological complete response (PCR) after neoadjuvant chemotherapy (34).

Trop-2 is also a key factor in the growth of TNBC tumors. Ning Li and colleagues analyzed Trop-2 expression in TNBC tissues and cells using the TCGA database, immunohistochemical staining, and qRT-PCR. They found that Trop-2 regulates TNBC cell proliferation and apoptosis through a Ca2+-dependent endoplasmic reticulum stress (ERS) signaling pathway, modulating these processes by influencing Ca2+ levels and ERS-related signaling pathways (35).

### 3.2 Trop-2 and breast cancer prognosis

The activation status of Trop-2 is also considered a new prognostic marker for breast cancer. Trop-2 is synthesized in the endoplasmic reticulum, transported to the Golgi apparatus for glycosylation, and then trafficked to the cell membrane. However, a significant amount of Trop-2 can also be retained intracellularly (36). Researchers conducted an immunohistochemical analysis of 702 human breast cancer cases (with a median follow-up time of 8 years) and found that surface membrane-localized and mature glycosylated Trop-2 is associated with poorer patient survival, while intracellular retention of Trop-2 is linked to fewer disease recurrences and better survival outcomes (37). Aslan et al. discovered that Trop-2 gene deletion and silencing can inhibit both *in vitro* and *in vivo* growth of TNBC cells. Trop-2 may contribute to oncogene-mediated metabolic reprogramming in TNBC by regulating a set of metabolic genes and oncogenes. Additionally, Trop-2 expression is associated with elevated levels of a five-gene metabolic signature (including TALDO1, GPI, LDHA, SHMT2, and ADK). Breast cancer patients with this five-gene metabolic signature have poorer overall survival and disease-free survival, making it a new and accurate predictor of breast cancer outcome (38).

Trop-2 plays a crucial role in tumor metastasis and promotes epithelial-to-mesenchymal transition (EMT), while E-cadherin is a key factor in cell adhesion and EMT. Wei Zhao and colleagues investigated the expression of Trop-2 and E-cadherin in BC and found that Trop-2+/E-cadherin- expression was significantly higher in BC tissues compared to adjacent matched tissues. Moreover, Trop-2+/E-cadherin- expression in BC is associated with lymph node status, metastasis, TNM staging, and ER-/PR-/HER2- expression. Importantly, BC patients with Trop-2+/E-cadherin- expression have poorer overall survival and prognosis (6).

### 3.3 Trop-2 expression and the efficacy of other therapies

Trop-2 expression may be linked to the efficacy of certain therapies. It has been reported that Trop-2 can contribute to the development of drug resistance, and its inhibition may mitigate this effect (39). As early as 2011, Oyama et al. discovered that Trop-2 overexpression can increase cytoplasmic Ca2+ levels, which in turn activates cyclic AMP response element-binding protein and the MAPK/ERK signaling pathway, promoting cell proliferation and leading to resistance to the selective estrogen receptor antagonist tamoxifen (40). In the same year, another study reported that Trop-2 overexpression contributes to resistance against trastuzumab monoclonal antibody therapy (41). Interestingly, chemotherapy itself may increase Trop-2 expression (38). In 2022, Jing Zhu et al. found that treatment with tamoxifen and irinotecan significantly elevated Trop-2 expression in breast ductal carcinoma cell lines (42). Therefore, Trop-2 expression not only impacts the development of drug resistance but also influences the efficacy of various treatments for breast cancer, highlighting an important area of concern.

## 4 Major drugs and clinical research

### 4.1 Sacituzumab Govitecan

SG is the first third-generation ADC targeting Trop-2 to receive market approval. It is currently the only ADC approved for use in advanced TNBC and hormone receptor-positive/human epidermal growth factor receptor 2-negative (HR+/HER2-) breast cancer. SG consists of the active metabolite of irinotecan, SN-38, conjugated via a cleavable linker (CL2A) to the humanized IgG1 monoclonal antibody HRS7, with a drug-to-antibody ratio (DAR) ranging from 7.5 to 8 (43, 44). SN-38 functions as a topoisomerase inhibitor, inducing DNA damage by inhibiting topoisomerase I, and exhibits cytotoxicity that is 100 to 1000 times greater than that of irinotecan (45).

#### 4.1.1 TNBC

Trop-2 is highly expressed in over 90% of TNBC cases, rendering it a promising therapeutic target (46). The IMMU-132-01 trial (NCT01631552), a Phase I/II study, was the first to evaluate the single-agent activity, tolerability, and safety of SG across various advanced cancers, including lung cancer, breast cancer, and urothelial carcinoma. This study encompassed 108 patients with advanced metastatic TNBC, demonstrating an objective response rate (ORR) of 33.3%, a clinical benefit rate (CBR) of 45.4%, a median duration of response (DoR) of 7.7 months, a median progression-free survival (PFS) of 5.5 months, and a median overall survival (OS) of 13.0 months (47). Based on these results, the U.S. Food and Drug Administration (FDA) granted accelerated approval for SG in April 2020 for patients with advanced metastatic TNBC who had previously received at least two lines of therapy (48).

Subsequently, the Phase III ASCENT trial (NCT02574455) further validated the efficacy of SG (49). This trial enrolled 468 patients with refractory, advanced TNBC who had received at least two prior treatments, with the objective of comparing the efficacy of SG against the physician’s choice of single-agent chemotherapy, such as eribulin, vinorelbine, capecitabine, or gemcitabine. The results showed that the SG group (n=235) had a statistically significant improvement in median PFS (5.6 vs. 1.7 months, hazard ratio [HR] 0.39, P<0.0001) and median OS (12.1 vs. 6.7 months, HR 0.48, P<0.0001) in comparison to the treatment of physician’s choice (TPC) group (n=233). The ORR was 35% for the SG group versus 5% for the TPC group. Additionally, the ASCENT trial evaluated efficacy based on Trop-2 expression levels (50). Patients with medium to high Trop-2 expression demonstrated comparable PFS (5.6 months vs. 6.9 months) and OS (14.9 months vs. 14.2 months) to those with low Trop-2 expression, who had relatively inferior PFS (2.7 months) and OS (9.3 months).

However, additional prospective trials are necessary to further validate the association between Trop-2 expression levels and treatment efficacy. Based on these findings, SG was approved in April 2021 for the treatment of unresectable locally advanced or metastatic TNBC patients who had previously undergone two or more systemic therapies (51). Furthermore, the Phase II NeoSTAR trial (NCT04230109) demonstrated that SG achieved a PCR rate of 30% in untreated TNBC patients with tumors ≥1 cm or any size with positive lymph nodes after four cycles of treatment (52). This underscores SG’s antitumor efficacy in the neoadjuvant setting and establishes it as the first ADC used in this context for TNBC.

TNBC is characterized by significant heterogeneity and a lack of well-defined molecular targets, which limits the effectiveness of targeted therapies observed in non-selective clinical trials. TNBC can be classified into four transcriptionally-based subtypes: (1) Luminal androgen receptor (LAR) subtype, characterized by androgen receptor signaling (23%); (2) Immunomodulatory (IM) subtype, defined by high expression of immune cell signaling and cytokine-mediated pathways (24%); (3) Basal-like immunosuppressed (BLIS) subtype, featuring upregulation of cell cycle processes, activation of DNA repair mechanisms, and downregulation of immune response genes (39%); and (4) Mesenchymal-like (MES) subtype, enriched in breast cancer stem cell pathways (15%) (53, 54). To address this challenge, the ongoing umbrella trial, Future-Trop-2, aims to evaluate the efficacy and safety of first-line Trop-2-targeted therapy in patients with locally advanced or metastatic TNBC, guided by molecular pathways. This study stratifies patients according to the four TNBC signaling pathways to assess the correlation between the efficacy of Trop-2 ADC and these molecular mechanisms, thus driving the development of more precise and personalized therapeutic strategies. Currently, multiple clinical trials are assessing the efficacy of SG in TNBC treatment, including its combination with immunotherapy for metastatic TNBC (e.g., NCT03424005, NCT04468061) and with PARP inhibitors for metastatic TNBC (e.g., NCT04039230) (55). Overall, SG has demonstrated significant improvements in ORR, PFS, and OS compared to standard chemotherapy regimens in patients with Trop-2-positive advanced TNBC.

Furthermore, SG demonstrates a relatively favorable safety profile, with the most frequently observed adverse effects being myelosuppression and diarrhea. Nonetheless, as follow-up duration increases, there is an observable trend toward a rise in SG-related adverse events. Approximately 60% of patients experience diarrhea, with around 10% of these cases classified as grade 3 or higher. This escalation in adverse events may be attributable to off-target toxicity resulting from premature cleavage of the drug-linker (51).

As research continues to advance, the potential applications of SG in neoadjuvant therapy, as a first-line treatment for advanced TNBC, and in combination with immunotherapy are expected to become increasingly apparent. Furthermore, the extension of follow-up periods is essential for comprehensive monitoring of SG-related adverse events.

#### 4.1.2 HR+/HER2-

SG has also demonstrated significant efficacy in patients with HR+/HER2- metastatic breast cancer. The Phase III clinical trial TROPiCS-02 (NCT03901339) enrolled 543 patients with endocrine-resistant and chemotherapy-resistant HR+/HER2- metastatic breast cancer, who had previously undergone endocrine therapy and treatment with CDK4/6 inhibitors. With a median follow-up period of 12.5 months, the trial results showed that the SG group (n=272) had substantial advantages over the TPC group (n=272) in terms of median PFS (5.5 vs. 4.0 months, HR=0.66, 95% confidence interval [CI]: 0.53–0.83, P<0.01), median OS (14.4 vs. 11.2 months, HR=0.79, 95% CI: 0.65–0.96, P=0.020), and ORR (21% vs. 14%, OR=1.63, 95% CI: 1.03–2.56, P=0.035) (56). Trop-2 is highly expressed in 95% of HR+/HER2- metastatic breast cancer patients. The study revealed that patients with varying levels of Trop-2 expression showed similar results in PFS and OS, indicating that SG consistently improves prognosis regardless of Trop-2 expression levels (57). Based on these findings, SG became the first Trop-2-targeted ADC to demonstrate dual benefits in PFS and OS for patients with endocrine-pretreated advanced breast cancer. In 2023, the U.S. FDA approved SG for use in patients with unresectable or metastatic HR+/HER2- breast cancer who have received at least two prior systemic therapies for metastatic disease, including endocrine therapy.

#### 4.1.3 HER2 low expression

Research has indicated that low HER-2 expression does not influence the efficacy of SG. In the TROPiCS-02 trial, 92% of participants exhibited HER2 IHC 0 or low HER2 expression. The baseline characteristics of these subgroups were comparable to those of the intent-to-treat population, and both subgroups demonstrated a benefit in terms of median progression-free survival (PFS) (HR, 0.72, P < 0.05; HR, 0.58, P < 0.001) (8). The most frequently reported treatment-related adverse events in both the SG and TPC groups included neutropenia (63% vs. 43%), diarrhea (59% vs. 12%), and nausea (57% vs. 26%) (57).

The safety profile of SG was consistent with previous studies, including the preliminary analysis of TROPiCS-02 and the ASCENT trial. However, there was one reported fatality due to septic shock secondary to neutropenic colitis. An exploratory study comparing safety across different genotypes revealed that patients with the UGT1A1 *28/*28 genotype experienced a higher incidence of grade ≥3 adverse events compared to those with the UGT1A1 *1/*28 and *1/*1 genotypes (15% vs. 9% and 10%, respectively) (56). Further research is warranted to confirm these findings and to provide more precise guidance for enhancing the safety of SG therapy.

Overall, the data substantiate the efficacy and safety of SG as a monotherapy. SG constitutes an effective treatment option for HR+/HER2- breast cancer patients, regardless of HER2 IHC 0 or low HER2 expression (56). Extensive investigations have evaluated SG across various molecular subtypes and stages of metastatic breast cancer, both as a standalone treatment and in combination with other therapies, as summarized in **Table 1**.

**Table 1:** Summary of representative clinical trials involving sacituzumab govitecan.

NCT number	Acronym	Phase	Combination	Patient Population	Recruitment Status
NCT01631552	IMMU-132-01	Ib/II	–	Epithelial cancer (TNBC, Non-TNBC)	Completed
NCT02574455	ASCENT	III	–	Refractory/​relapsed metastatic TNBC	Completed
NCT03901339	TROPiCS-02	III	–	HR+/​HER2- metastatic breast cancer	Completed
NCT05840211	ASCENT-07	III	–	HR+/ HER2- inoperable, locally advanced, or metastatic breast cancer and have received endocrine therapy	Recruiting
NCT04595565	SASCIA	III	–	Patients with HER2-negative breast cancer with residual disease after neoadjuvant chemotherapy	Active, not recruiting
NCT04647916	–	II	–	HER2-Negative Breast Cancer and Brain Metastases	Recruiting
NCT05552001	ISIdE	III	–	Locally advanced or metastatic triple-negative breast cancer	Recruiting
NCT04230109	NeoSTAR	II	Cohort A2 and C: pembrolizumab	Cohort A1 and A2: localized TNBC Cohort B1: HR+ breast cancer Cohort C: HER2- inflammatory breast cancer	Recruiting
NCT04039230	–	Ib/II	Talazoparib	Metastatic breast cancer	Recruiting
NCT05008510	VERU-111	II	Sabizabulin	Metastatic breast cancer	Withdrawn^a^
NCT05143229	ASSET	I	Alpelisib	Metastatic or locally recurrent HER2-negative breast cancer	Recruiting
NCT06081244	ADAPT-TN-III	II	Pembrolizumab	Low-risk, triple-negative early breast cancer	Not yet recruiting
NCT04434040	ASPRIA	II	Atezolizumab	TNBC	Active, not recruiting
NCT04448886	Saci-IO HR+	II	Pembrolizumab	Locally advanced or metastatic HR-positive, HER2-negative, PD-L1 positive breast cancer	Active, not recruiting
NCT04468061	Saci-IO TNBC	II	Pembrolizumab	Locally advanced or metastatic TNBC, PD-L1 negative	Recruiting
NCT03424005	Morpheus-panBC	Ib/II	Atezolizumab	Metastatic or unresectable locally advanced triple-negative or HR-positive/​ HER2-negative breast cancer	Recruiting
NCT05675579	–	II	Pembrolizumab	Immunochemotherapy-resistant early-stage TNBC	Recruiting
NCT05633654	ASCENT-05/​AFT-65 OptimICE-RD/​NSABP B-63	III	Pembrolizumab	Patients with triple negative breast cancer who have residual invasive disease after surgery and neoadjuvant therapy	Recruiting
NCT05382286	ASCENT-04	III	Pembrolizumab	Patients with previously untreated, locally advanced inoperable or metastatic triple-negative breast cancer	Recruiting
NCT03971409	InCITe	II	Avelumab	Stage IV or unresectable, recurrent triple negative breast cancer	Recruiting
NCT04927884	–	Ib/II	Chemoimmunotherapy (cyclophosphamide, N-803, and PD-L1 t-haNK)	Advanced triple-negative breast cancer after prior therapies	Terminated^b^
NCT06100874	SATEEN	II	Trastuzumab	HER2+ metastatic breast cancer	Recruiting
NCT05928780	FUTURE-Trop2	I/II	SHR3680/ SHR1210/ SHR3162/ VEGFRI	Locally advanced or metastatic triple-negative breast cancer	Not yet recruiting

Data collected until August 11, 2024. Data were obtained from clinicaltrials.gov.

a: Decided to halt and will potentially reopen in the future.

b: Due to low enrollment.

### 4.2 Datopotamab Deruxtecan

Dato-DXd is an ADC targeting Trop-2, comprising of a humanized anti-Trop-2 IgG1 monoclonal antibody, a cleavable peptide linker, and the DNA topoisomerase I inhibitor, exatecan mesylate (DXd). Compared to SG, Dato-DXd exhibits a longer half-life (approximately 45.1 ± 13.9 hours), which extends drug exposure in tumors and may enhance its anti-tumor activity and response durability. Preclinical studies indicate that DXd demonstrates potent anti-tumor activity in TNBC by inducing DNA damage and apoptosis, and it is more effective than SN-38 in tumor suppression (58, 59). Relevant studies are summarized in **Table 2**.

**Table 2:** Summary of representative clinical trials involving datopotamab deruxtecan.

NCT number	Acronym	Phase	Combination	Patient Population	Recruitment Status
NCT03401385	TROPION-PanTumor01	I	–	Advanced solid tumors (HR+/HER2- breast cancer)	Recruiting
NCT05104866	TROPION-Breast01	III	–	Participants with inoperable or metastatic HR+/HER2- breast cancer who have been treated with one or two prior lines of systemic chemotherapy	Active, not recruiting
NCT05374512	TROPION-Breast02	III	–	Patients with locally recurrent inoperable or metastatic triple-negative breast cancer, who are not candidates for PD-1/​PD-L1 inhibitor therapy	Active, not recruiting
NCT06176261	–	II	–	HER2-negative metastatic breast cancer.	Recruiting
NCT05866432	TUXEDO-2	II	–	Triple-negative breast cancer patients with newly diagnosed or progressing brain metastases.	Recruiting
NCT03742102	BEGONIA	Ib/II	Durvalumab	Advanced/unresectable or metastatic triple-negative breast cancer	Active, not recruiting
NCT06112379	TROPION-Breast04	III	Durvalumab	Neoadjuvant/​adjuvant treatment of triple-negative or hormone receptor-low/​HER2-negative breast cancer	Recruiting
NCT05629585	TROPION-Breast03	III	Durvalumab	Stage I-III triple-negative breast cancer without pathological complete response following neoadjuvant therapy	Recruiting
NCT06103864	TROPION-Breast05	III	Durvalumab	Patients with PD-L1 positive locally recurrent inoperable or metastatic triple-negative breast cancer	Recruiting
NCT04644068	PETRA	I/IIa	AZD5305	Advanced solid malignancies (Breast Cancer)	Recruiting

Data collected until August 11, 2024. Data were obtained from clinicaltrials.gov.

#### 4.2.1 HR+/HER2-

Dato-DXd has demonstrated promising efficacy in patients with HR+/HER2- metastatic breast cancer. The ongoing Phase I TROPION-PanTumor01 study (NCT03401385) is assessing the efficacy and safety of Dato-DXd in this patient cohort. Recent data indicate that Dato-DXd demonstrates significant anti-tumor activity in heavily pretreated patients with a manageable safety profile. Among the 41 patients enrolled, the majority (95%) had been previously treated with CDK4/6 inhibitors. The results revealed an ORR of 27%, with 11 patients achieving a partial response [PR]) and a disease control rate (DCR) of 85%. The median PFS as assessed by Blinded Independent Central Review (BICR) was 8.3 months (95% CI, 5.5–11.1 months), indicating durable tumor responses. The most common adverse events included oral mucositis (83%), nausea (56%), fatigue (46%), and alopecia (37%), with the majority classified as grade 1-2. Treatment discontinuation occurred in five patients due to corneal inflammation, corneal lesions, oral mucositis, and pneumonia. Oral mucositis remain a notable adverse effect of Dato-DXd, highlighting the need for careful management of oral mucosal toxicity to prevent mucosal inflammation (60).

In the Phase III TROPION-Breast01 trial (NCT05104866), the efficacy and safety of Dato-DXd were compared with investigator’s choice of standard single-agent chemotherapy (including eribulin, vinorelbine, gemcitabine, or capecitabine) in patients with HR+/HER2- breast cancer who had previously received one or two lines of systemic chemotherapy and were either ineligible for surgery or had metastatic disease (61). With a median follow-up of 10.8 months, the findings demonstrated that Dato-DXd significantly enhanced median PFS in comparison to chemotherapy (6.9 months vs. 4.9 months; HR=0.63; 95% CI, 0.52–0.76; p<0.0001). Subgroup analysis indicated that the efficacy of Dato-DXd was consistent regardless of prior CDK4/6 inhibitor treatment. The observed difference in ORR between the Dato-DXd group (36.4%) and the chemotherapy group (2.9%) underscores its clinical superiority. Although OS data are not yet mature, the Dato-DXd group shows a trend towards benefit (HR=0.84; 95% CI, 0.62–1.14). Regarding safety, the median treatment duration was 6.7 months for the Dato-DXd group compared to 4.1 months for the chemotherapy group. The incidence of treatment-related adverse events (TRAEs) was slightly higher in the Dato-DXd group (94% vs. 86%). However, the incidence of grade ≥3 TRAEs (21% vs. 45%) and the proportion of dose reductions (21% vs. 30%) or treatment interruptions (12% vs. 25%) due to TRAEs were lower in the Dato-DXd group. The most frequently reported TRAEs in the Dato-DXd group were nausea (51%) and oral mucositis (50%), with most events being grade 1-2 (62).

Overall, Dato-DXd significantly prolonged PFS in patients with HR+/HER2- advanced breast cancer who exhibited resistance to endocrine therapy. Furthermore, it demonstrated excellent anti-tumor efficacy in 82% of patients who had previously been treated with CDK4/6 inhibitors. Additionally, the TRAEs associated with Dato-DXd were generally mild and manageable, suggesting a controllable safety profile and favorable patient tolerance.

#### 4.2.2 TNBC

In the phase I TROPION-PanTumor01 trial (NCT03401385), the efficacy and safety of Dato-DXd were evaluated in patients with metastatic TNBC who had progressed after multiple lines of standard treatments (63). The results indicated that among 44 evaluable patients, the BICR reported an ORR of 32%, including one CR and 13 PR. The DCR reached 80%, with a median PFS of 4.3 months and a median OS of 12.9 months (64). During treatment, the most frequently observed grade ≥3 adverse events were oral mucositis (11%), fatigue (7%), and lymphopenia (7%) (60).

The chemotherapeutic effect of topoisomerase I inhibitors can enhance the efficacy of immunotherapy. Therefore, the combination of Dato-DXd with immune checkpoint inhibitors is being explored as a promising therapeutic strategy. In the phase Ib/II BEGONIA study (NCT03742102), Dato-DXd was administered in conjunction with the PD-L1 inhibitor durvalumab as a first-line treatment for patients with unresectable, locally advanced, or metastatic TNBC, demonstrating sustained therapeutic benefits (65). As of February 2, 2023, a total of 62 patients had been treated with the Dato-DXd and durvalumab combination, with 29 patients (47%) remaining on treatment. The combination demonstrated an ORR of 79% (49/62; 95% CI, 66.8-88.3), including 6 patients (10%) with CR and 43 patients (69%) with PR. The median PFS was 13.8 months, and the median DoR was 15.5 months, with anti-tumor activity observed regardless of PD-L1 expression levels. In terms of safety, the most frequently reported adverse events were nausea and oral mucositis, with 23% of patients experiencing serious adverse events; however, no treatment-related deaths were reported (66).

These promising results not only offer a new therapeutic option for patients with advanced TNBC but also underscore the extensive potential of combining Dato-DXd with immunotherapy in the treatment of breast cancer. Presently, the phase III TROPION-Breast04 trial (NCT06112379) is further evaluating the efficacy and safety of Dato-DXd and durvalumab in neoadjuvant or adjuvant settings for TNBC or HR-low/HER2- breast cancer (67). Additional phase III clinical trials for Dato-DXd in the field of TNBC are also underway, including the TROPION-Breast02 trial (NCT05374512) (64)evaluating first-line monotherapy for advanced TNBC, and the TROPION-Breast03 trial (NCT05629585) (68) assessing adjuvant intensification therapy for TNBC. Moreover, two phase II clinical trials are actively recruiting participants to investigate the efficacy of Dato-DXd in the treatment of brain metastases originating from breast cancer. (NCT06176261, NCT05866432).

The safety profile of Dato-DXd is generally manageable, with a limited number of patients discontinuing treatment due to adverse reactions (58). Overall, Dato-DXd has exhibited significant potential in the treatment of TNBC, particularly among patients expressing Trop-2. Its efficacy and safety have received preliminary validation, suggesting that it may become a valuable therapeutic option.

### 4.3 SKB264 (MK-2870)

SKB264 is an innovative Trop-2-targeted ADC featuring the same humanized antibody HRS7 as SG. SKB264 utilizes a cleavable linker containing 2-(methylsulfonyl) pyrimidine to conjugate with a novel topoisomerase I inhibitor, the Belotecan derivative KL610023 (T030) (69). The drug-antibody ratio (DAR) of SKB264 is similar to that of SG (7.4 vs. 7.6); however, the conjugation method used in SKB264 is more stable, potentially reducing side effects. Specifically, SKB264 utilizes a nucleophilic aromatic substitution reaction between methanesulfonate and thiol, thereby circumventing the complications observed with SG, where thiol-maleimide linkers interact with albumin’s free thiol groups, resulting in linker-payload detachment. This methodology enhances stability and optimizes the safety-efficacy profile (70). Preclinical investigations have shown that SKB264 exhibits a markedly extended half-life in murine models compared to SG (57 hours versus 14 hours), suggesting improved stability and prolonged pharmacological action (71).

#### 4.3.1 TNBC

In the treatment of locally advanced or metastatic TNBC, SKB264 has exhibited significant anti-tumor activity. In the Phase II A264 expansion trial (NCT04152499), with a median follow-up duration of 9.6 months, the confirmed ORR was 40% (22/55), and the DCR was 80% (44/55). In patients with high Trop-2 expression, the confirmed ORR reached up to 55%. The median PFS was 5.7 months (95% CI: 3.9-7.6) (11).

Regarding safety, 55.9% of patients experienced TRAEs of grade 3 or higher, with the most prevalent being neutropenia (23.7%), anemia (20.3%), and thrombocytopenia (16.9%). 15.2% of patients required dose reductions due to adverse events, and 6.8% discontinued treatment due to TRAEs. Notably, there were no treatment-related fatalities or instances of interstitial lung disease reported. These findings suggest that SKB264 demonstrates substantial anti-tumor efficacy and a manageable safety profile in patients with heavily pretreated locally advanced or metastatic TNBC (72).

The ongoing Phase III randomized controlled trial, OptiTROP-Breast01 (NCT05347134), is assessing the efficacy of SKB264 in patients with locally advanced, recurrent, or metastatic TNBC who have experienced failure with at least two prior lines of therapy. Preliminary findings indicate that SKB264 substantially enhances median progression-free survival (PFS) compared to chemotherapy (6.7 months vs. 2.5 months; HR = 0.32, 95% CI: 0.22–0.44; P < 0.001), and also confers a significant benefit in OS (HR = 0.53; 95% CI: 0.36–0.78; P = 0.0005). Additionally, the ORR has markedly improved (45.4% vs. 12%). The safety profile of SKB264 remains manageable (73). Based on these promising results, SKB264 has demonstrated the most favorable median PFS among existing treatments for post-line TNBC. Consequently, it has been submitted for regulatory approval in China for use in patients with unresectable locally advanced or metastatic TNBC who have previously undergone at least two systemic therapies, including one for the advanced or metastatic stage.

#### 4.3.2 HR+/HER2-

In an I/II phase basket study (NCT04152499), SKB264 demonstrated notable efficacy and safety in patients with HR+/HER2- metastatic breast cancer who had previously undergone at least one line of chemotherapy (65). With a median follow-up duration of 8.2 months, the study reported a median PFS of 11.1 months, an ORR of 36.8%, and a DCR of 89.5%. These results highlight that SKB264 offers significant efficacy benefits over traditional chemotherapy for HR+/HER2- metastatic breast cancer patients. The study also explored efficacy across different patient subgroups and found that SKB264 was effective across various groups, including those with low or no HER2 expression, those resistant to endocrine therapy, and those previously treated with CDK4/6 inhibitors. These findings indicate that SKB264 may offer significant benefits to a heterogeneous patient population, thereby underscoring its potential role in personalized treatment strategies.

In terms of safety, the most frequently observed grade ≥3 TRAEs were neutropenia (36.6%), leukopenia (22%), anemia (14.6%), and thrombocytopenia (9.8%). Importantly, the study reported no treatment discontinuations or fatalities attributable to TRAEs, suggesting a favorable tolerance profile and manageable safety (74).

The application of SKB264 in HR+/HER2- breast cancer is presently under further investigation through two ongoing Phase III clinical trials. The first trial (NCT06081959) targets patients who have previously undergone at least one line of chemotherapy, while the second trial (NCT06279364) focuses on chemotherapy-naive patients. Both studies are designed to evaluate the efficacy and safety of SKB264 in comparison to the investigator’s choice of chemotherapy. Relevant studies are summarized in **Table 3**.

**Table 3:** Summary of representative clinical trials involving SKB264.

NCT number	Acronym	Phase	Combination	Patient Population	Recruitment Status
NCT04152499	A264	I/II	–	Advanced unresectable/​metastatic solid tumors, refractory to standard therapies (HR+/ HER2- breast cancer)	Recruiting
NCT05347134	OptiTROP-Breast01	III	–	Locally advanced, recurrent or metastatic triple-negative breast cancer	Active, not recruiting
NCT06081959	–	III	–	Locally advanced, recurrent or metastatic HR+/​HER2- breast cancer	Recruiting
NCT06279364	–	III	–	Unresectable recurrent or metastatic triple-negative breast cancer	Recruiting
NCT05445908	–	II	KL-A167	Recurrent or metastatic HER2-negative breast cancer	Recruiting

Data collected until August 11, 2024. Data were obtained from clinicaltrials.gov.

The distinctive structural design of SKB264, coupled with its optimal balance between anti-tumor efficacy and safety, positions it as a promising candidate for targeting Trop-2 in breast cancer, specifically in HR+/HER2- patients. Consequently, SKB264 holds potential to become an integral component of future therapeutic strategies.

### 4.4 PF-06664178 (RN927C)

PF-06664178 is an ADC featuring a humanized anti-Trop-2 IgG1 antibody (RN926) conjugated with Aur0101, a derivative of auristatin and a microtubule inhibitor (75). Upon binding to the extracellular portion of Trop-2 on the cell surface, the ADC is internalized into the lysosome where proteolytic cleavage releases the Aur0101 payload, thereby exerting its antitumor effects. Preclinical studies have demonstrated that RN927C exhibits potent *in vitro* cytotoxicity against Trop-2-positive tumor cell lines. *In vivo* investigations have demonstrated that RN927C exhibits substantial antitumor activity in patient-derived xenograft models, including those of TNBC, lung cancer, and ovarian cancer, outperforming standard treatments such as paclitaxel and gemcitabine (76).

In 2014, a Phase I clinical trial was initiated to determine the maximum tolerated dose (MTD) of RN927C and to assess its safety and tolerability in patients with locally advanced or metastatic solid tumors. However, the trial findings indicated that the toxicity associated with high doses of RN927C was disproportionate to its antitumor efficacy. Notably, the dose-limiting toxicities (DLTs) observed were attributed to the stable linker and potent payload, which resulted in an inadequate therapeutic window. These toxicity issues led to the premature termination of the study (77).

Despite RN927C exhibiting substantial antitumor efficacy in preclinical investigations, its clinical progression was impeded by severe dose-limiting toxicities. This experience highlights the critical balance between a stable linker and a potent payload in the development of ADCs. The challenges encountered with RN927C provide valuable insights for the design of future Trop-2-targeted ADCs and underscore the importance of safety evaluation in ADC drug development.

### 4.5 SHR-A1921

SHR-A1921 is a novel Trop-2-targeted ADC composed of a proprietary IgG1 monoclonal antibody conjugated with a topoisomerase I inhibitor, SHR9265, via a cleavable linker. SHR9265, a novel derivative of exatecan, exhibits enhanced lipophilicity and cell permeability, and has demonstrated remarkable stability and efficacy in both *in vitro* and *in vivo* studies (78).

In a first-in-human (FIH) Phase I clinical trial conducted in China (NCT05154604), SHR-A1921 demonstrated promising efficacy and a favorable safety profile in patients with various advanced solid tumors who had previously failed multiple treatments. Preliminary results indicate an ORR of 33.3% (10/30; 95% CI 17.3-52.8) and a DCR of 80.0% (24/30; 95% CI 61.4-92.3). The most common TRAEs were nausea (71.1%), oral mucositis (65.8%), and anemia (42.1%). Notably, 31.6% of patients experienced grade ≥3 TRAEs, with oral mucositis being the most frequent at 18.4% (79).

The ongoing clinical trial aims to further evaluate the efficacy of SHR-A1921 across various dosing regimens and cancer types, with the objective of continuing to explore its therapeutic potential in the treatment of advanced solid tumors.

### 4.6 DB-1305

DB-1305 represents a novel ADC targeting Trop-2, comprising an anti-Trop-2 antibody linked to a new topoisomerase I inhibitor, P1021, through a cleavable linker incorporating a maleimide tetrapeptide. Preclinical investigations have revealed that DB-1305 exerts significant antitumor efficacy in models of TNBC, colorectal cancer, and lung cancer. Compared to DS-1062, DB-1305 has shown equivalent or superior tumor cell proliferation inhibition and possesses an improved safety profile (80).

The findings indicate that DB-1305 exhibits considerable potential for the treatment of TNBC. Presently, DB-1305 is under investigation in an ongoing Phase I/IIa multicenter, non-randomized clinical trial (NCT05438329). This trial is designed to evaluate the safety, tolerability, pharmacokinetics, and antitumor efficacy of DB-1305 in patients with advanced solid tumors.

## 5 Combination therapies

**Figure 3:**
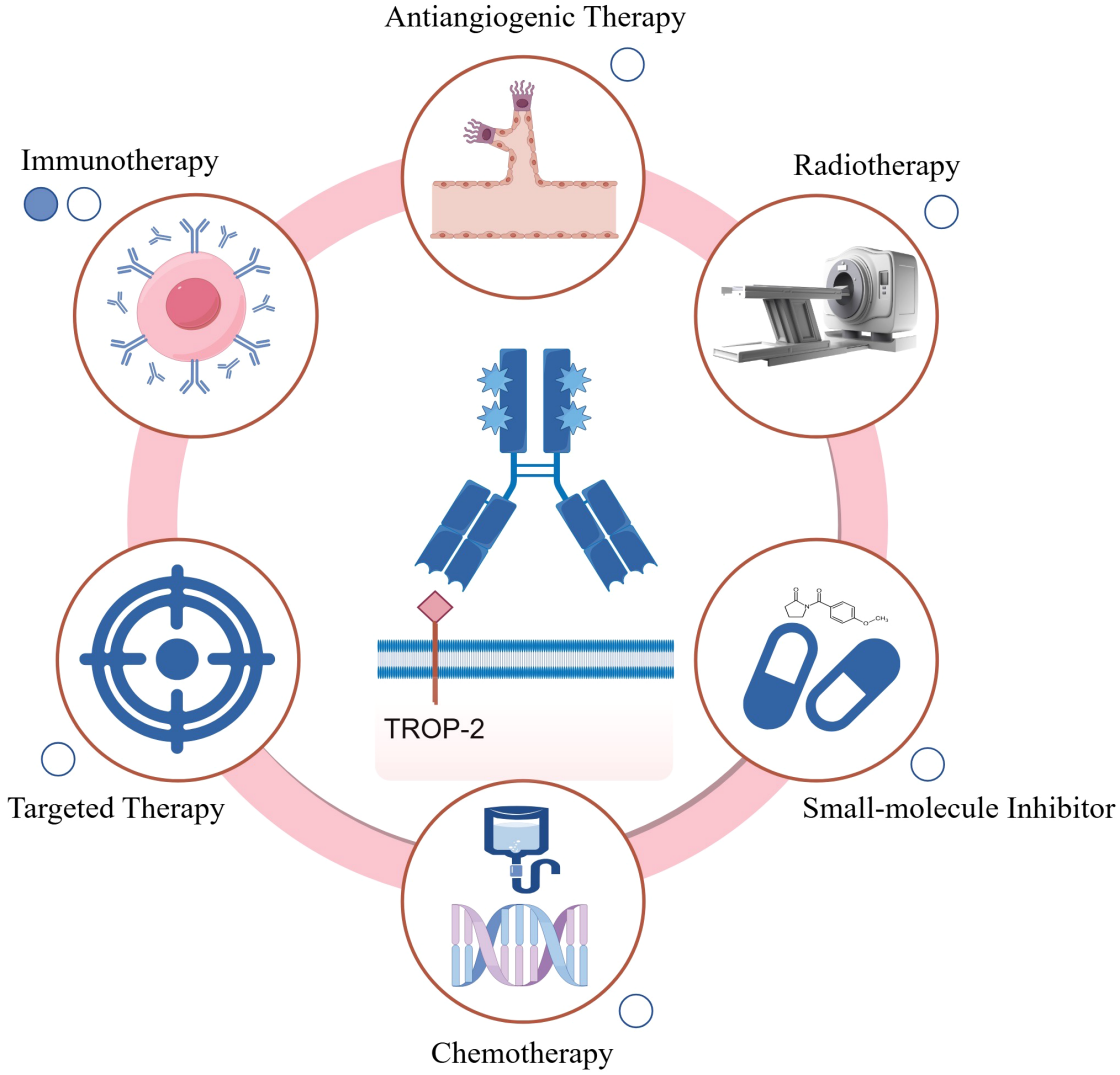
Summary of Trop-2-targeted ADC combination therapies. Available published data (including abstracts) are represented by solid circles, while ongoing studies are indicated by hollow circles. Chemotherapy: Trop-2-targeted ADC combined with chemotherapy can exert synergistic effects, enhancing cytotoxicity towards rapidly dividing cells, such as bone marrow cells, potentially leading to bone marrow suppression; Targeted therapy: The combination of Trop-2 ADC with targeted therapies can block multiple oncogenic pathways or achieve dual inhibition of a single pathway, thereby more effectively suppressing downstream signaling; Anti-angiogenic therapy: Anti-angiogenic agents normalize the tumor vasculature, potentially improving the intratumoral delivery of Trop-2 ADCs; Radiotherapy: The combination of Trop-2 ADC with radiotherapy may enhance antitumor activity through radiosensitization, though potential cumulative toxicity needs further evaluation; Small-molecule inhibitors: Trop-2 ADC combined with small-molecule inhibitors targets multiple pathways simultaneously, enhancing tumor growth suppression; Immunotherapy: Trop-2 ADC can promote immune surveillance by reprogramming macrophages and activating dendritic and T cells, enhancing the efficacy of immune checkpoint inhibitors. This combination also helps optimize the tumor microenvironment for improved therapeutic outcomes. The figure was generated using Figdraw (www.figdraw.com).

Currently, the combination of Trop-2-targeted ADCs with other antitumor therapies, including chemotherapy agents, small molecule inhibitors, immunotherapies, anti-angiogenic drugs, and radiotherapy, is actively being explored (**Figure 3**). Existing studies demonstrate that these combination strategies hold substantial promise for enhancing therapeutic efficacy, overcoming resistance, and improving patient prognosis.

### 5.1 Combination with chemotherapy and targeted therapies

#### 5.1.1 Combination with chemotherapy

ADCs in combination with chemotherapy predominantly target the cell cycle, with a particular emphasis on the S phase. This combination enhances therapeutic efficacy by inducing G2/M phase arrest through the action of DNA-damaging agents. Common chemotherapeutic agents, including platinum compounds and topoisomerase inhibitors, can be effectively used alongside microtubule inhibitors to achieve superior therapeutic outcomes (81). Furthermore, certain chemotherapeutic drugs have the capacity to modulate the expression of surface antigens targeted by ADCs. For example, gemcitabine has been demonstrated to upregulate the expression of HER2.

Nonetheless, the timing of administration and the potential cumulative toxicity of the drugs remain pivotal considerations. ADCs characterized by a high DAR and cleavable linkers may present an elevated risk of off-target toxicity. Consequently, it is imperative to meticulously evaluate potential pharmacokinetic interactions and payload toxicity when selecting combination therapies.

In two prior Phase 1b/2a studies, the combination of T-DM1 with docetaxel or paclitaxel was evaluated in patients with HER2-positive metastatic breast cancer. Although some clinical benefits were observed, nearly half of the patients necessitated dose reduction or discontinuation due to adverse effects (82, 83). Dato-DXd has exhibited a more favorable safety profile in solid tumor trials, suggesting its potential as an ideal partner for combination therapy. Nonetheless, the efficacy of Trop-2-targeted ADCs in conjunction with chemotherapy for breast cancer treatment still warrants further clinical investigation and validation.

#### 5.1.2 Combination with targeted therapies

The integration of ADCs with targeted therapies represents a strategic approach to overcoming resistance commonly associated with monotherapy. This method facilitates the simultaneous inhibition of multiple oncogenic pathways or the dual blockade of a specific pathway, thereby enhancing the suppression of downstream signaling cascades. Furthermore, these combinations can augment the efficacy of ADCs by modulating surface antigen expression, consequently increasing the susceptibility of tumors with low antigen expression to treatment.

Tyrosine kinase inhibitors (TKIs), as small-molecule targeted agents, have demonstrated clinical efficacy when combined with ADCs. In the TEAL trial (NCT02073487), the combination of T-DM1, lapatinib (a TKI), and nab-paclitaxel exhibited superior efficacy compared to standard therapy in the neoadjuvant treatment of early-stage HER2-positive breast cancer, without significant differences in safety profiles (84). Subsequent studies have also shown that the combination of T-DM1 with tucatinib (a TKI) exhibits robust antitumor activity and manageable toxicity in patients with advanced HER2-positive breast cancer (85).

Furthermore, the ongoing SATEEN trial (NCT06100874) aims to evaluate the safety and efficacy of SG in combination with trastuzumab for patients with metastatic HER2-positive breast cancer. This study is anticipated to provide significant insights into the therapeutic potential of SG in non-HER2 receptor-negative breast cancer (refer to **Table 1**).

### 5.2 Combination with small molecule inhibitors

#### 5.2.1 PARP inhibitors

Poly (ADP-ribose) polymerase (PARP) constitutes a family of enzymes integral to the processes of DNA damage repair and apoptosis. The inhibition of PARP impedes the repair of damaged DNA, resulting in the accumulation of DNA double-strand breaks. In cancers characterized by BRCA1/2 mutations, this inhibition increases the susceptibility of cancer cells to PARP inhibitors, culminating in cell death (86). Empirical studies have shown that ADCs carrying topoisomerase I inhibitor payloads can synergistically inhibit the proliferation of breast cancer tumor cells when used in conjunction with PARP inhibitors (87). However, there may also be a greater cytotoxic effect on rapidly dividing cells, such as bone marrow cells, which can lead to severe bone marrow suppression.

Irrespective of BRCA1/2 status, the combination of SG with PARP inhibitors (including olaparib, rucaparib, and talazoparib) markedly augments DNA damage in TNBC cells relative to monotherapy. In murine models of both BRCA1/2-mutant and wild-type TNBC, combination therapy has demonstrated superior antitumor efficacy compared to single-agent treatment, with favorable tolerability and an absence of severe hematologic toxicity. These results indicate that the combination of SG with PARP inhibitors may provide enhanced clinical benefits for the treatment of TNBC (88).

Currently, a Phase I/II clinical trial is evaluating the efficacy of SG in combination with talazoparib (a PARP inhibitor) in patients with metastatic TNBC (NCT04039230) (89). Additionally, preclinical studies have shown that the combination of Dato-DXd with AZD5305 (a PARP1 inhibitor) enhances cytotoxicity in TNBC cell lines compared to monotherapy. The benefits of combination therapy have been observed across all doses, irrespective of homologous recombination deficiency (HRD) status, and were well tolerated *in vivo* (89). These findings further support the potential of Dato-DXd as both a monotherapy and in combination with AZD5305 for the treatment of various advanced solid tumors (NCT05489211, NCT04644068).

#### 5.2.2 PI3K inhibitors

The phosphatidylinositol-3-kinase (PI3K) pathway is an essential signaling cascade downstream of receptor tyrosine kinases (RTKs), orchestrating a multitude of cellular processes including growth, proliferation, differentiation, and apoptosis. Consequently, this pathway is pivotal in tumorigenesis and cancer progression. Mutations in the phosphatidylinositol-4,5-bisphosphate 3-kinase catalytic subunit alpha (PIK3CA) gene are present in approximately 40% of patients with HR+/HER2- breast cancer and are correlated with poor prognosis in advanced stages of the disease (91).

Drawing upon the findings from the SOLAR-1 study, the U.S. FDA has granted approval for alpelisib, a PI3K inhibitor, for the treatment of HR+/HER2- advanced breast cancer in patients harboring PIK3CA mutations (92). In this context, the ongoing Phase I ASSET trial (NCT05143229) seeks to establish the recommended Phase II dose of SG in conjunction with alpelisib for patients with metastatic or locally recurrent HER2-negative breast cancer, while also assessing the pharmacokinetics and ORR associated with this combination therapy. It is noteworthy that Alpelisib directly interferes with insulin action by inhibiting the PI3K signaling pathway, resulting in insulin resistance and hyperglycemia. When used in combination therapy, gastrointestinal adverse effects caused by SG may exacerbate this phenomenon (93).

### 5.2.3 Combination with other small molecule inhibitors

The exploration of Trop-2-targeted ADCs in combination with small molecule inhibitors is gaining momentum, with numerous clinical studies underway. These include microtubule inhibitors (e.g., sabizabulin, NCT05008510), ATR inhibitors (e.g., berzosertib, NCT02595931), CDK4/6 inhibitors (e.g., trilaciclib, NCT05113966), and MCL1 inhibitors (e.g., GS-9716, NCT05006794). As shown in **Table 4**, several of these agents have demonstrated promising preclinical efficacy, warranting further clinical exploration.

**Table 4:** Summary of clinical trials for other combination therapies.

Drug name	NCT number	Phase	Combination	Patient Population	Recruitment Status
Sacituzumab Govitecan	NCT05008510	II	Sabizabulin	Metastatic triple negative breast cancer	Withdrawn^a^
Irinotecan Hydrochloride	NCT02595931	I	Berzosertib	Patients with solid tumors that are metastatic or cannot be removed by surgery	Active, not recruiting
Sacituzumab Govitecan	NCT05113966	II	Trilaciclib	Patients with unresectable, locally advanced or metastatic TNBC who received at least 2 prior treatments, at least 1 in the metastatic setting	Active, not recruiting
Sacituzumab Govitecan	NCT05006794	I	GS-9716	Advanced solid malignancies	Recruiting
Sacituzumab Govitecan	NCT06238921	II	Zimberelimab and stereotactic radiation (SRS)	Metastatic triple negative breast cancer with brain metastases	Not yet recruiting

Data collected until August 11, 2024. Data were obtained from clinicaltrials.gov.

a: Decided to halt and will potentially reopen in the future.

### 5.3 Combination with immunotherapy

ADCs interact with both tumor and immune cells through mechanisms such as immunogenic cell death (ICD), antibody-dependent cellular cytotoxicity (ADCC), and dendritic cell activation, providing potential synergistic effects with immunotherapies. Clinical research is progressively investigating combination strategies involving Trop-2-targeted ADCs and immune checkpoint inhibitors, specifically anti-PD-1/PD-L1 and anti-CTLA-4 antibodies (94).

Upon binding to their specific target antigens on cancer cells and subsequent internalization, ADCs release cytotoxic payloads that induce ICD. This process results in the release of damage-associated molecular patterns (DAMPs) within the tumor microenvironment (TME), which are subsequently recognized by immature dendritic cells. The cytotoxic payload further facilitates the maturation of dendritic cells, leading to the activation of CD8+ T cells in the lymph nodes. These activated T cells are then capable of infiltrating the tumor, recognizing, and attacking cancer cells (95, 96).

Studies have demonstrated that HER2-targeting ADCs exhibit synergistic effects when combined with ICIs, enhancing the efficacy of immunotherapy in both *in vitro* and *in vivo* settings (97). Similarly, Trop-2-targeted ADCs have shown promising potential when used in combination with immunosuppressive agents. For example, SG enhances tumor sensitivity to anti-PD-1 therapy by recruiting natural killer (NK) or CD8+ T cells to infiltrate the TME, where they secrete interferon-gamma and granzyme B to mediate tumor cell cytotoxicity.

In the BEGONIA study (NCT03742102), the combination of Dato-DXd with a PD-L1 inhibitor exhibited significant efficacy and manageable toxicity in patients with advanced or metastatic TNBC (66). These results indicate that the combination of Trop-2-targeted ADCs with ICIs holds substantial promise. Nonetheless, additional clinical trials are warranted to confirm these potential benefits (refer to **Tables 2**, **3**).

### 5.4 Other potential combination strategies

#### 5.4.1 Combination with anti-angiogenic agents

Tumor angiogenesis is regulated by a balance between pro-angiogenic and anti-angiogenic factors, which are synthesized by both malignant and non-malignant cells through complex signaling pathways. Anti-tumor angiogenesis strategies frequently employ pharmacological agents that target vascular endothelial growth factor (VEGF) to impede its binding to receptors, thereby inhibiting the formation of blood vessels that support tumor growth. Inhibition of VEGF can result in the degradation of existing tumor vasculature, a reduction in vascular permeability, and diminished oxygen delivery to the tumor. Furthermore, the inhibition of VEGF may normalize the residual tumor vasculature, thereby ameliorating the interstitial pressure within the tumor and facilitating the delivery of chemotherapeutic agents. This process can further inhibit tumor angiogenesis. Anti-angiogenic agents and ADCs have distinct but complementary antitumor mechanisms, suggesting their potential to produce synergistic effects (98, 99).

Prior research has demonstrated that bevacizumab, an anti-angiogenic agent, can enhance the permeability of paclitaxel, thereby augmenting its anti-tumor efficacy. Furthermore, preclinical investigations in ovarian cancer have demonstrated that the combination of ADCs with VEGF inhibitors significantly enhances anti-tumor efficacy (100). While experimental data on the combination of Trop-2-targeted ADCs with anti-angiogenic agents are currently lacking, preclinical models have indicated that the active metabolite of SG, SN-38, has the potential to reduce VEGF levels. This indicates that the integration of SG with anti-angiogenic agents could potentially enhance anti-tumor efficacy. Additionally, it is essential to consider the vascular inhibitory effects of anti-angiogenic agents, which may increase the risk of bleeding and thrombosis. Therefore, monitoring for cardiovascular adverse effects when using these agents in combination therapy warrants careful attention. Nevertheless, this therapeutic approach necessitates additional research to substantiate its effectiveness and to formulate more tailored treatment protocols based on specific tumor characteristics and Trop-2 expression levels.

#### 5.4.2 Combination with radiotherapy

Radiotherapy induces tumor cell apoptosis and facilitates the release of tumor-associated antigens, thereby promoting the activation and proliferation of tumor-specific T cells. Nonetheless, tumors frequently exhibit mechanisms of immune evasion and immune modulation, which can attenuate the efficacy of radiotherapy as a monotherapy. Integrating radiotherapy with immunotherapy has the potential to circumvent these limitations and generate a synergistic therapeutic effect in oncological treatment.

Preclinical investigations have demonstrated that ADCs can induce immunogenic cell death and selectively enhance the radiosensitivity of tumor cells (101, 102). Notably, the ongoing Phase II PRAG 3.0 study (NCT05115500) is assessing the efficacy of combining RC48-ADC, a novel HER2-targeting ADC, with high-dose fractionated radiotherapy (HFRT), PD-1/PD-L1 inhibitors, and sequential administration of GM-CSF and IL-2 in the treatment of HER2-positive advanced solid tumors. Preliminary findings suggest that this combination therapy is associated with manageable safety profiles and promising efficacy, thereby offering a novel exploratory strategy for precision combination therapy (66, 103).

Similarly, Trop-2-targeted ADCs exhibit promising potential when used in conjunction with radiotherapy. An ongoing Phase I/II clinical trial (NCT06238921) is evaluating the safety and efficacy of SG in combination with zimberelimab and stereotactic radiosurgery (SRS) compared to SG monotherapy in patients with brain metastases originating from triple-negative breast cancer. The outcomes of this study are anticipated to provide further validation for the combined application of Trop-2-targeted ADCs with radiotherapy and immunotherapy.

Trop-2-targeted ADCs demonstrate significant therapeutic potential; however, to effectively translate this potential into clinical applications, future research must focus on optimization and rigorous assessment of potential toxicities. Inappropriate dosing or timing of combination therapies can lead to severe toxic reactions, necessitating the development of predictive models for toxicity. Such models should leverage individual patient data to assess the risk of adverse effects associated with combination therapies and establish more detailed toxicity monitoring standards for various drug combinations. For instance, when used in conjunction with immune checkpoint inhibitors, immune-related toxicities (such as immune-mediated inflammation, hepatitis, and pneumonia) may be more pronounced, particularly in patients with pre-existing robust immune responses. Similarly, the systemic toxicity of small molecule inhibitors (including hepatic, renal, and cardiac toxicities) may be exacerbated when used in combination with Trop-2-targeted ADCs.

## 6 Future directions and challenges

The expression level of Trop-2 varies significantly among breast cancer patients and is inconsistent across different subtypes. This variability is closely associated with tumor aggressiveness, prognosis, and responsiveness to therapeutic interventions (2). Currently, no standardized method exists to accurately quantify Trop-2 expression, leading to potential errors in patient selection during clinical trials, which may compromise the reliability of research findings. Although Trop-2 is highly expressed in breast tumors, it is also present at low levels in certain normal tissues, potentially resulting in unavoidable “target-related” toxicity. Current clinical studies face several challenges; many clinical trials—especially early-phase studies—enroll relatively few patients, making it difficult to achieve sufficient statistical power to validate efficacy. Furthermore, data regarding the long-term efficacy and safety of Trop-2-targeted ADCs remain limited, particularly concerning their effects on recurrence rates and patient survival. Following the FDA’s approval of SG for the treatment of advanced and metastatic triple-negative and hormone receptor-positive/HER2-negative breast cancer, additional novel Trop-2-targeted ADCs are currently under active development. These novel ADCs integrate advancements in antibody selection, payload optimization, and linker design, with the objective of achieving significant improvements in safety and efficacy.

Future research should focus on developing more precise biomarker assays to better identify patients who are sensitive to the efficacy of Trop-2-targeted ADCs. This approach may include a combination of techniques such as IHC, flow cytometry, and molecular diagnostics. Additionally, exploring the quantitative relationship between Trop-2 expression levels and treatment efficacy will help determine which patients are most likely to benefit from therapy and whether dose adjustments are necessary for specific individuals. Regarding ADC optimization, enhancing antibody specificity is essential to accurately target Trop-2 on tumor cells while minimizing expression in non-malignant tissues, thereby reducing off-target toxicity. Potential toxicity stacking could be predicted by simulating the multi-target effects of drug combinations using gene editing or *in vitro* 3D tumor culture models. Furthermore, the risk of side effects from combination therapies can be anticipated based on individual patient data, utilizing big data analysis tools such as machine learning. However, similar to most cytotoxic agents, the duration of objective response and clinical benefit from monotherapy is limited due to the development of resistance mechanisms.

Consequently, exploring potential combination therapies and identifying synergistic antitumor effects have become research priorities. Some combination treatments have demonstrated promising antitumor activity and safety in preclinical studies, such as Dato-DXd combined with AZD5305 and SG combined with Talazoparib (89, 90). Future clinical trials hold the potential to introduce novel treatment modalities for refractory or resistant breast cancer.

However, the cumulative cytotoxicity associated with combination therapies remains a substantial challenge. Future research priorities will include optimizing patient selection, refining ADC components, and developing more effective combination strategies to overcome resistance and improve patient outcomes. Emerging studies and ongoing trials will be crucial in shaping the future landscape of Trop-2-targeting therapies and advancing personalized treatment approaches.
